# Supplementary material for: National and subnational burden of leukemia and its risk factors, 1990–2019: Results from the Global Burden of Disease study 2019
Source: PLoS One. 2023 Nov 30;18(11):e0287917. doi: 10.1371/journal.pone.0287917 (PMC10688854; doi:10.1371/journal.pone.0287917)
Supplement: S1 Table — (PDF) [file pone.0287917.s001.pdf]

Supplementary Table 1. Age-standardized rate of leukemia's burden by sex and year at provincial level with percent change

Province	Measure	Age-standardized rate (per 100,000)						% Change (1990 to 2019)		
		1990			2019					
		Both	Female	Male	Both	Female	Male	Both	Female	Male
Alborz	Incidence	8.7 (6 to 11.7)	8.7 (5.4 to 12.4)	8.8 (5.9 to 12.3)	7.4 (6.1 to 9)	6.3 (4.9 to 8.1)	8.5 (5.8 to 11)	-14.9 (-40.7 to 34.5)	-27.5 (-55.2 to 31.8)	-3.6 (-36.2 to 55.7)
	Prevalence	36.1 (22.3 to 53.7)	38.3 (20.2 to 62.2)	34.2 (20.3 to 52.3)	35.1 (26.6 to 43.8)	31 (22.5 to 42.5)	39.1 (23.6 to 52)	-2.7 (-39.3 to 80)	-18.9 (-54.7 to 74.9)	14.2 (-32.2 to 108.4)
	Deaths	6.3 (4.4 to 7.9)	5.8 (3.6 to 7.8)	6.8 (4.7 to 9.2)	4.6 (3.7 to 5.6)	3.6 (2.9 to 4.5)	5.5 (4 to 7.3)	-27.1 (-45.1 to 9.9)	-37.8 (-55.5 to 0.9)	-19.2 (-44.6 to 24)
	DALYs	224.8 (153.2 to 297.2)	217.9 (126.6 to 306.3)	231.8 (155.8 to 318.1)	147.9 (119.1 to 176.5)	119.5 (93.7 to 146.9)	175.2 (128.2 to 223.7)	-34.2 (-53.1 to 3.1)	-45.2 (-63.1 to 2.5)	-24.4 (-49.2 to 23.8)
	YLLs	220.5 (149.9 to 291.6)	213.5 (123.7 to 300)	227.5 (152.8 to 312.6)	143.8 (116 to 171.9)	116 (90.6 to 143.4)	170.6 (125.3 to 218.6)	-34.8 (-53.5 to 2.3)	-45.7 (-63.5 to 1.8)	-25 (-50 to 23.2)
	YLDs	4.3 (2.5 to 6.4)	4.3 (2.3 to 6.9)	4.3 (2.5 to 6.7)	4.1 (2.7 to 5.6)	3.5 (2.2 to 5)	4.6 (2.7 to 6.7)	-5.8 (-36.3 to 56.9)	-19.8 (-51.8 to 50)	7.2 (-32 to 87.3)
Ardebil	Incidence	15.2 (8.6 to 23.2)	15.8 (7.3 to 27.5)	14.6 (7 to 22.6)	9.4 (6.1 to 11.5)	7.4 (4.8 to 9.1)	11.6 (6.3 to 15)	-38.2 (-59.3 to -1.7)	-53.4 (-73.4 to -11.2)	-20.7 (-46.4 to 35)
	Prevalence	66.5 (28.2 to 116.8)	76.2 (25.7 to 156.8)	57 (20.7 to 97.9)	34.9 (20.5 to 45.2)	29.4 (16.9 to 39)	40.7 (19.4 to 55.1)	-47.5 (-70.3 to 12.4)	-61.4 (-82 to 2.8)	-28.6 (-59.1 to 52.1)
	Deaths	9.8 (6.3 to 13.3)	9 (5.2 to 12.7)	10.6 (5.7 to 15.3)	6.9 (4.7 to 8.4)	4.8 (3.2 to 5.8)	9 (5.2 to 11.7)	-30.3 (-46.3 to -3.5)	-46.3 (-61.8 to -21.1)	-15 (-39.2 to 34.8)
	DALYs	427.4 (244.5 to 620)	427.5 (199.6 to 673.4)	425 (182.4 to 656.8)	223.8 (143.3 to 276.3)	173.8 (106.1 to 208.7)	275.1 (148.7 to 360)	-47.6 (-63.1 to -22.9)	-59.3 (-73.7 to -29.2)	-35.3 (-56.3 to 17.2)
	YLLs	420.3 (241 to 612)	420.2 (197.1 to 659.4)	418.2 (179.2 to 647.1)	219.4 (140.3 to 270.8)	170.4 (104.4 to 203.9)	269.7 (145.9 to 352.5)	-47.8 (-63.2 to -23.1)	-59.5 (-73.7 to -29.5)	-35.5 (-56.6 to 17.1)
	YLDs	7 (3.4 to 11.8)	7.3 (3 to 13.9)	6.7 (2.8 to 11.6)	4.4 (2.5 to 6.6)	3.5 (2 to 5.1)	5.4 (2.7 to 8.3)	-37.2 (-60.3 to 12.1)	-52.4 (-74.9 to -0.9)	-19.8 (-49 to 50.7)

Province	Measure	Age-standardized rate (per 100,000)						% Change (1990 to 2019)		
		1990			2019					
		Both	Female	Male	Both	Female	Male	Both	Female	Male
Bushehr	Incidence	12.2 (7.4 to 15.9)	13 (6.4 to 17.7)	11.5 (6.5 to 15.7)	10.1 (6.7 to 12.4)	9.4 (5 to 11.6)	11 (6.7 to 13.9)	-17 (-37.2 to 23.7)	-27.8 (-50.1 to 14.1)	-4.4 (-35.5 to 56.6)
	Prevalence	48.3 (25.6 to 70)	56.4 (23.7 to 87)	40.7 (19.9 to 59.3)	43.9 (26.2 to 56.3)	43.8 (21.7 to 57.9)	44.6 (23.9 to 59.3)	-9.1 (-38.5 to 64.8)	-22.2 (-53.9 to 53.1)	9.6 (-34.6 to 102.5)
	Deaths	8.9 (5.6 to 11.3)	8.7 (4.6 to 11.7)	9.2 (5.4 to 12.4)	6.7 (4.7 to 8)	5.6 (3.3 to 6.8)	7.9 (5.2 to 9.9)	-24.2 (-40.2 to 6.8)	-35.1 (-51.4 to -7.7)	-13.8 (-39.3 to 35.4)
	DALYs	323.9 (196.3 to 423.8)	343 (155.9 to 453.8)	305.8 (164.4 to 420.4)	207.3 (135 to 248.5)	192 (102.3 to 232.5)	224.2 (138.8 to 281.4)	-36 (-51.7 to -7.3)	-44 (-59.7 to -13)	-26.7 (-49.7 to 20.5)
	YLLs	318.2 (192.8 to 415.1)	336.8 (152.8 to 445.6)	300.5 (161.3 to 414.3)	201.9 (131.4 to 241.3)	186.9 (99.6 to 226.5)	218.5 (136.1 to 274.9)	-36.5 (-52.2 to -7.9)	-44.5 (-59.9 to -13.5)	-27.3 (-50.3 to 19.9)
	YLDs	5.8 (3 to 8.8)	6.2 (2.8 to 9.7)	5.4 (2.7 to 8.4)	5.4 (3.1 to 7.8)	5 (2.4 to 7.3)	5.8 (3 to 8.7)	-7.2 (-31.9 to 51.8)	-19.5 (-46.5 to 37.1)	7.3 (-30.8 to 85.9)
Chahar Mahaal and Bakhtiari	Incidence	10.9 (6.7 to 14.9)	9.9 (5.5 to 14.3)	11.9 (6.6 to 16.9)	8.9 (5.2 to 11.8)	7 (3.8 to 9.7)	11 (5.7 to 15.1)	-18.3 (-41.9 to 25.7)	-29.5 (-57.5 to 20.8)	-7.8 (-40.2 to 48.4)
	Prevalence	41.6 (21.1 to 62.4)	41.2 (18 to 70.1)	41.9 (19.6 to 63.3)	36 (18.7 to 51.2)	31.1 (15.5 to 48.1)	41.3 (19.4 to 60.9)	-13.5 (-46.5 to 62.9)	-24.5 (-64.2 to 70)	-1.4 (-44.9 to 85.2)
	Deaths	8 (5.2 to 10.1)	6.5 (3.9 to 8.5)	9.4 (5.7 to 13.1)	5.7 (3.6 to 7.3)	3.8 (2.4 to 5)	7.8 (4.1 to 10.5)	-28 (-46.5 to 0.1)	-41.2 (-59.3 to -16.3)	-17.4 (-42.1 to 27)
	DALYs	299.5 (185 to 396.2)	267.6 (148 to 366.2)	328.3 (174.4 to 465.7)	185.2 (109.2 to 243.3)	139.6 (78.1 to 186.4)	232.3 (116.8 to 313.6)	-38.2 (-56.5 to -10)	-47.9 (-67.4 to -17.4)	-29.3 (-52.8 to 13.8)
	YLLs	294.6 (182.8 to 391.8)	263.2 (145.7 to 359.9)	323 (171.5 to 459)	180.9 (106.5 to 237.1)	136.2 (76.8 to 181.8)	227.1 (113.7 to 306.9)	-38.6 (-56.8 to -10.8)	-48.3 (-67.6 to -18)	-29.7 (-53.3 to 13)
	YLDs	4.8 (2.6 to 7.3)	4.4 (2.2 to 7.2)	5.3 (2.7 to 8.3)	4.2 (2.1 to 6.4)	3.4 (1.5 to 5.3)	5.1 (2.3 to 8.2)	-12.6 (-40 to 45.4)	-22.7 (-56.5 to 47.2)	-3.1 (-38.7 to 65.3)

Province	Measure	Age-standardized rate (per 100,000)						% Change (1990 to 2019)		
		1990			2019					
		Both	Female	Male	Both	Female	Male	Both	Female	Male
East Azarbayejan	Incidence	14.4 (9.1 to 19)	14.9 (7.9 to 20.4)	13.9 (6.9 to 19.8)	10 (6.8 to 12.2)	8.7 (5.7 to 11)	11.2 (6.5 to 14.7)	-30.9 (-49.1 to 3.2)	-41.5 (-61.3 to 0)	-19.4 (-43.7 to 29.9)
	Prevalence	58 (32.1 to 83.7)	67.1 (28.7 to 107.2)	49.2 (20.4 to 75.7)	39.4 (24.4 to 50.3)	36.9 (21.9 to 50.1)	41.9 (21.5 to 57.2)	-32.1 (-56.1 to 20.1)	-45 (-69.5 to 19)	-14.7 (-47 to 58.5)
	Deaths	10 (6.9 to 12.7)	9.2 (5.6 to 11.9)	10.9 (6.2 to 14.9)	7.2 (5 to 8.7)	5.8 (4 to 7.1)	8.6 (5.2 to 11.1)	-28.7 (-44.2 to -2.4)	-37.2 (-54.3 to -8.9)	-21.2 (-43.2 to 18)
	DALYs	418.4 (254 to 564.7)	418.6 (197.4 to 578.8)	417 (195.7 to 607.1)	240 (153.8 to 296.9)	205.7 (128.9 to 255.3)	274 (153.7 to 365)	-42.6 (-57.3 to -17.5)	-50.9 (-66.3 to -16.2)	-34.3 (-55.2 to 8.7)
	YLLs	411.7 (250.4 to 555.8)	411.5 (193.1 to 569.6)	410.6 (192.5 to 598.6)	235 (151.6 to 291.6)	201.2 (126.2 to 250.6)	268.5 (150.3 to 356.2)	-42.9 (-57.6 to -17.9)	-51.1 (-66.6 to -16.6)	-34.6 (-55.6 to 8.5)
	YLDs	6.7 (3.6 to 10.2)	7.1 (3.4 to 11)	6.3 (2.9 to 10.4)	5 (2.9 to 7.1)	4.4 (2.6 to 6.5)	5.5 (2.8 to 8.2)	-25.5 (-47.9 to 18)	-37.1 (-60.6 to 15.6)	-12.9 (-42.4 to 49.1)
Fars	Incidence	12.2 (7.8 to 16)	10.8 (6.4 to 14.4)	13.6 (7.2 to 19.1)	11 (7.1 to 14)	9 (5.7 to 11.8)	12.9 (6.9 to 17.5)	-10 (-32.6 to 32.4)	-16.6 (-43.1 to 32.5)	-5.3 (-35 to 49.6)
	Prevalence	46.9 (27.5 to 64.3)	46 (23.2 to 65.5)	47.9 (21.9 to 71.1)	48.4 (28.6 to 64.9)	42.4 (24 to 59.1)	54.3 (25.1 to 77.7)	3.2 (-27.6 to 75.3)	-7.8 (-42.2 to 70.1)	13.2 (-28.8 to 101.8)
	Deaths	9.1 (5.9 to 12)	7.2 (4.3 to 9.8)	11 (6.1 to 15.3)	7.2 (4.8 to 9)	5.4 (3.4 to 6.7)	8.9 (5.1 to 11.8)	-21 (-41.7 to 9.4)	-25.8 (-46.3 to 7.8)	-18.8 (-44.6 to 25.7)
	DALYs	340.6 (210.1 to 456.6)	294.1 (156 to 399.3)	385.6 (189.1 to 548.1)	253.6 (164.4 to 318.7)	201 (117.9 to 255.3)	305 (162 to 410.1)	-25.5 (-45.2 to 5.8)	-31.7 (-52.8 to 5.1)	-20.9 (-46.5 to 26.5)
	YLLs	334.9 (206.6 to 450.1)	289 (152.7 to 394.2)	379.3 (184.8 to 539.9)	247.8 (161.2 to 312)	196.2 (115 to 248.3)	298.4 (158.6 to 401.4)	-26 (-45.6 to 5.5)	-32.1 (-53.4 to 4.8)	-21.3 (-47.1 to 26.3)
	YLDs	5.7 (3.2 to 8.5)	5.1 (2.8 to 7.6)	6.3 (3 to 10)	5.7 (3.2 to 8.5)	4.8 (2.6 to 7.1)	6.6 (3 to 10.3)	0.4 (-26.1 to 57.7)	-6.6 (-38.3 to 56.7)	5.3 (-29.8 to 77.6)

Province	Measure	Age-standardized rate (per 100,000)						% Change (1990 to 2019)		
		1990			2019					
		Both	Female	Male	Both	Female	Male	Both	Female	Male
Gilan	Incidence	10.5 (7.1 to 13.8)	10.3 (6.2 to 14.4)	10.9 (6.8 to 14.9)	9.3 (6.4 to 11.4)	8.3 (5.2 to 10.3)	10.3 (6.2 to 13.4)	-12 (-35.2 to 27.7)	-19.6 (-45.4 to 24.8)	-6.1 (-34.5 to 41.4)
	Prevalence	38.6 (22.5 to 56.1)	41.8 (21.1 to 65.6)	35.7 (19.7 to 52.9)	39.5 (24.5 to 50.5)	37.3 (20.8 to 49.6)	41.7 (21.6 to 57)	2.4 (-32.5 to 73.2)	-10.9 (-47.2 to 68.3)	16.8 (-25.8 to 101.4)
	Deaths	7.8 (5.5 to 9.8)	7 (4.5 to 9.2)	8.8 (5.8 to 11.7)	6.2 (4.5 to 7.5)	5.3 (3.5 to 6.4)	7.2 (4.6 to 9.3)	-19.7 (-36.5 to 8.6)	-24.4 (-45.2 to 8.9)	-17.7 (-41.1 to 21.5)
	DALYs	286.2 (187.3 to 374.8)	272.3 (147.9 to 372.9)	301.8 (174.9 to 417.6)	209.6 (140.5 to 257)	177.1 (105.5 to 219.2)	242.1 (145.1 to 315.6)	-26.7 (-44.9 to 6)	-35 (-55.8 to 3.7)	-19.8 (-45.2 to 25.5)
	YLLs	280.8 (183.3 to 369.5)	266.9 (144.5 to 367.8)	296.3 (172.3 to 409.8)	204.8 (137.6 to 252.1)	172.7 (103.2 to 214.4)	236.8 (140.3 to 309.6)	-27.1 (-45.2 to 5.8)	-35.3 (-56.3 to 3.3)	-20.1 (-45.5 to 24.9)
	YLDs	5.4 (3 to 7.9)	5.4 (2.8 to 8.2)	5.5 (2.9 to 8.5)	4.8 (2.9 to 6.8)	4.4 (2.4 to 6.3)	5.2 (2.7 to 7.9)	-10.4 (-36.7 to 35.9)	-18 (-47.2 to 32.8)	-4.5 (-37 to 50.9)
Golestan	Incidence	10.2 (7.4 to 12.9)	10.1 (6.6 to 13.7)	10.4 (6.7 to 13.9)	7.9 (6.5 to 9.2)	6.6 (5.2 to 7.9)	9.2 (7 to 11.4)	-22.6 (-40 to 12.8)	-34.3 (-53.8 to 0.8)	-11.6 (-36.4 to 37.4)
	Prevalence	36.4 (22.6 to 50.8)	39.3 (20.9 to 61)	33.6 (17.8 to 48.4)	26.6 (20.5 to 32.3)	23.1 (16.5 to 30.1)	30.3 (19.9 to 39)	-26.8 (-49.3 to 27.4)	-41.3 (-65.2 to 14.3)	-9.8 (-40.6 to 55.4)
	Deaths	7.7 (5.9 to 9.3)	6.9 (4.9 to 8.7)	8.5 (5.8 to 11.2)	6 (5.1 to 7)	4.8 (3.8 to 5.6)	7.3 (5.6 to 9.2)	-21.6 (-38.1 to 8.7)	-31.1 (-48.9 to -2.4)	-14.5 (-38.4 to 26.2)
	DALYs	308.3 (227.9 to 394.5)	304.9 (187.6 to 409.2)	311.9 (176.5 to 425.6)	208.1 (170.6 to 242.7)	172.3 (128.7 to 202.1)	244.8 (176.7 to 298.5)	-32.5 (-47.2 to -5)	-43.5 (-59 to -12.3)	-21.5 (-43.1 to 23.3)
	YLLs	303.9 (225 to 389.5)	300.5 (184 to 403.8)	307.4 (174 to 420)	204.6 (167.4 to 238.7)	169.4 (126.6 to 199.3)	240.6 (173.9 to 293.9)	-32.7 (-47.4 to -5.5)	-43.6 (-59.3 to -12.4)	-21.7 (-43.4 to 22.8)
	YLDs	4.4 (2.7 to 6.4)	4.4 (2.4 to 6.7)	4.6 (2.6 to 6.9)	3.5 (2.4 to 4.9)	2.9 (1.9 to 4.1)	4.2 (2.7 to 5.9)	-20.4 (-40.4 to 24.2)	-33.2 (-55 to 10.3)	-8.7 (-36.2 to 49.2)

Province	Measure	Age-standardized rate (per 100,000)						% Change (1990 to 2019)		
		1990			2019					
		Both	Female	Male	Both	Female	Male	Both	Female	Male
Hamadan	Incidence	13.9 (8.9 to 19)	14.4 (7.3 to 21.3)	13.4 (7.3 to 19.1)	9.9 (6.7 to 12.2)	8.6 (5 to 10.8)	11.2 (6.7 to 14.9)	-29 (-49.2 to 6.3)	-40.4 (-62 to -0.7)	-16.6 (-44 to 32.6)
	Prevalence	59.2 (31.9 to 88.4)	68 (27.5 to 112.9)	50.8 (23.1 to 78.1)	39.3 (24.3 to 50.9)	36.1 (19.8 to 49.2)	42.5 (22.2 to 59.3)	-33.7 (-57.9 to 23.2)	-46.8 (-71.4 to 16.6)	-16.2 (-49.8 to 52.1)
	Deaths	9.4 (6.6 to 12.1)	8.6 (5.1 to 11.3)	10.2 (6.3 to 14.3)	6.9 (4.7 to 8.4)	5.5 (3.4 to 6.9)	8.2 (5 to 10.7)	-27 (-44.7 to -1)	-35.3 (-52.6 to -5.6)	-19.5 (-44.5 to 18.1)
	DALYs	399.5 (261.4 to 543.2)	388.3 (188.1 to 550.9)	408.9 (202.9 to 594.2)	247.2 (159.9 to 308.8)	205.7 (113.7 to 259.1)	288.6 (167.4 to 385.4)	-38.1 (-54.9 to -11.9)	-47 (-64.2 to -12.8)	-29.4 (-52.7 to 10.8)
	YLLs	392.9 (257 to 535.4)	381.4 (183.3 to 541.8)	402.7 (199.1 to 583.4)	242.3 (156.5 to 303.3)	201.4 (111.7 to 254.3)	283.1 (164.3 to 377.7)	-38.3 (-55.1 to -12.2)	-47.2 (-64.3 to -12.6)	-29.7 (-53 to 10.3)
	YLDs	6.6 (3.6 to 10.2)	6.9 (3.3 to 11.1)	6.2 (3 to 10)	4.9 (2.9 to 7.1)	4.3 (2.2 to 6.3)	5.5 (2.8 to 8.3)	-25.7 (-48.6 to 19.6)	-37.9 (-62.3 to 8.8)	-12.2 (-45 to 48.8)
Hormozgan	Incidence	9.1 (5.6 to 11.9)	8.4 (4.7 to 11.9)	9.8 (5.9 to 13.2)	6.7 (5.3 to 8.1)	5.1 (4 to 6.6)	8.2 (5.8 to 10.3)	-26.4 (-48.9 to 31.4)	-38.7 (-60.7 to 17.5)	-15.7 (-44.3 to 50.2)
	Prevalence	33.8 (18.3 to 48.4)	35.3 (17.7 to 57.1)	32.2 (17.4 to 47.2)	25.9 (18.9 to 33)	21.1 (15.4 to 28.9)	30.7 (19 to 40.4)	-23.4 (-52.6 to 59.6)	-40.3 (-68.2 to 44.3)	-4.7 (-43.1 to 94.6)
	Deaths	6.9 (4.5 to 8.8)	5.7 (3.5 to 7.6)	8.2 (5.2 to 11)	4.8 (3.9 to 5.8)	3.5 (2.8 to 4.3)	6.1 (4.5 to 7.8)	-30.7 (-47.7 to 16)	-39.2 (-57.1 to 1.9)	-24.8 (-49.1 to 27.8)
	DALYs	266.5 (152 to 354.9)	241.3 (122.4 to 338.8)	288.7 (157.8 to 405.8)	166.8 (134.2 to 202.5)	118.9 (91.8 to 147)	214 (155 to 270.3)	-37.4 (-56.2 to 16)	-50.7 (-68 to 1.4)	-25.9 (-51.9 to 35.1)
	YLLs	262.3 (149.6 to 350)	237.3 (120.4 to 333.9)	284.2 (154.7 to 399)	163.5 (132 to 198.4)	116.3 (89.4 to 143.9)	210 (152.7 to 265.6)	-37.6 (-56.4 to 16)	-51 (-68.3 to 1.4)	-26.1 (-52.2 to 35.2)
	YLDs	4.2 (2.2 to 6.1)	3.9 (1.9 to 6)	4.4 (2.4 to 6.8)	3.3 (2.2 to 4.6)	2.6 (1.6 to 3.8)	4 (2.4 to 5.8)	-20.9 (-46.7 to 50.3)	-34.1 (-59.4 to 37.7)	-9.4 (-41.7 to 74.6)

Province	Measure	Age-standardized rate (per 100,000)						% Change (1990 to 2019)		
		1990			2019					
		Both	Female	Male	Both	Female	Male	Both	Female	Male
Ilam	Incidence	10.6 (6.6 to 14.2)	8.5 (5.5 to 12.1)	12.2 (6.8 to 16.9)	8.5 (6 to 10.4)	5.9 (4.8 to 7.3)	10.9 (6.4 to 13.9)	-19.3 (-42.5 to 24.1)	-30 (-54 to 13.4)	-10.9 (-41.8 to 41.4)
	Prevalence	43.3 (19.6 to 64.1)	37.4 (16 to 62.8)	47.9 (21.4 to 70.9)	32.3 (20.6 to 41.8)	24.3 (17.2 to 33.1)	39.7 (21.5 to 53.1)	-25.4 (-52.5 to 46.7)	-35 (-65.1 to 48.7)	-17 (-54.6 to 61.2)
	Deaths	7.5 (5.2 to 9.5)	5.4 (3.9 to 7.1)	9.2 (5.6 to 12.5)	6 (4.4 to 7.2)	3.8 (3.2 to 4.5)	8 (5 to 10.2)	-19.6 (-38.1 to 16)	-29.8 (-49.6 to -0.3)	-12.7 (-38.3 to 32.7)
	DALYs	284.2 (188.3 to 376.3)	229.6 (155.3 to 303.2)	328.6 (173 to 467.6)	195.2 (137.3 to 236.1)	130 (104.6 to 155)	256.6 (151.8 to 329.3)	-31.3 (-49.2 to 2.2)	-43.4 (-59.4 to -11)	-21.9 (-46.6 to 24.8)
	YLLs	279.3 (185 to 370.6)	225.6 (152.1 to 298)	322.8 (170.3 to 461.9)	191.2 (134.7 to 231.6)	127.1 (102.8 to 152)	251.6 (148.3 to 323.3)	-31.5 (-49.4 to 1.6)	-43.7 (-59.5 to -11.3)	-22.1 (-46.6 to 24.6)
	YLDs	4.9 (2.5 to 7.6)	4 (2 to 6.4)	5.7 (2.7 to 9)	4 (2.4 to 5.7)	2.9 (1.8 to 4.1)	5 (2.7 to 7.4)	-18.9 (-43.5 to 38.7)	-27.4 (-54.2 to 32.8)	-12.1 (-46.3 to 55)
Isfahan	Incidence	10.9 (6.5 to 14.4)	10.5 (5.7 to 14.3)	11.4 (6.1 to 16.3)	10 (6.1 to 12.5)	8.8 (4.9 to 11.4)	11.2 (5.6 to 15.2)	-7.9 (-30.7 to 31.5)	-15.8 (-42 to 34.7)	-1.7 (-34.2 to 54.6)
	Prevalence	42.9 (23.8 to 60.6)	45.1 (21.8 to 67.6)	41 (19.9 to 62.5)	43.5 (24.2 to 56.5)	40.8 (19.9 to 56.3)	46.2 (20.6 to 65.7)	1.5 (-28.7 to 62.6)	-9.5 (-44.3 to 62.7)	12.7 (-29.3 to 96.7)
	Deaths	8 (4.6 to 10.3)	7 (4 to 9.2)	9 (5 to 12.6)	6.7 (4.1 to 8.3)	5.4 (3.3 to 6.8)	7.9 (3.9 to 10.5)	-15.9 (-36.3 to 20.8)	-22.3 (-44.2 to 19.1)	-12.4 (-41.5 to 35.8)
	DALYs	297.8 (167.2 to 395)	276.1 (140.6 to 373.5)	319.3 (164.3 to 459.3)	220.3 (129.1 to 274.6)	186.4 (103.1 to 235.2)	253.3 (123.3 to 338.5)	-26 (-45.4 to 8.2)	-32.5 (-53 to 6.1)	-20.7 (-48.2 to 28.8)
	YLLs	292.6 (164.3 to 388.2)	271.1 (138.1 to 368.6)	314 (161.5 to 452.2)	215.1 (126.1 to 268.6)	181.7 (100.6 to 229.7)	247.5 (121.5 to 331.9)	-26.5 (-45.9 to 7.9)	-33 (-53.5 to 6)	-21.2 (-48.5 to 29)
	YLDs	5.1 (2.6 to 7.7)	5 (2.4 to 7.5)	5.3 (2.5 to 8.6)	5.2 (2.7 to 7.6)	4.7 (2.3 to 7)	5.7 (2.6 to 9)	1.9 (-25.2 to 54.6)	-6.7 (-37.5 to 54.3)	8.7 (-30.4 to 83.8)

Province	Measure	Age-standardized rate (per 100,000)						% Change (1990 to 2019)		
		1990			2019					
		Both	Female	Male	Both	Female	Male	Both	Female	Male
Kerman	Incidence	10.5 (7.3 to 13.3)	9.4 (6.4 to 12.4)	11.7 (6.8 to 15.6)	8.1 (6.1 to 10)	7 (5 to 8.8)	9.2 (6.1 to 11.9)	-22.6 (-42.3 to 13.3)	-25.2 (-48.2 to 16.6)	-21 (-47.4 to 28.1)
	Prevalence	37.6 (23.6 to 51)	37.6 (21.6 to 56.2)	37.7 (19.5 to 54)	31.5 (21.5 to 40.6)	29.2 (18.5 to 39)	33.9 (18.8 to 46.9)	-16.1 (-43.7 to 45.5)	-22.4 (-52.9 to 44.8)	-10.1 (-48.6 to 71.1)
	Deaths	8.1 (6 to 10)	6.5 (4.8 to 8.4)	9.7 (6.4 to 12.9)	5.9 (4.6 to 6.9)	4.7 (3.5 to 5.7)	7.1 (4.9 to 8.7)	-27.4 (-43.2 to 1.8)	-28.2 (-46.9 to 2.5)	-27.6 (-49 to 11.2)
	DALYs	302.7 (214 to 388.7)	265.6 (171.4 to 355.1)	338.5 (188.9 to 463.1)	203.7 (153.2 to 246.3)	173.4 (119.1 to 215.9)	233.3 (157.4 to 291.4)	-32.7 (-49.8 to -1.8)	-34.7 (-54.9 to 3.1)	-31.1 (-53.3 to 10.5)
	YLLs	298 (210.4 to 382.7)	261.2 (167.7 to 350)	333.2 (185.9 to 456.6)	199.7 (150.2 to 240.9)	169.9 (116.9 to 211.5)	228.8 (154 to 285.9)	-33 (-50 to -2.2)	-35 (-55 to 2.6)	-31.3 (-53.3 to 10.6)
	YLDs	4.8 (2.9 to 6.8)	4.3 (2.5 to 6.4)	5.3 (2.9 to 7.9)	4 (2.5 to 5.7)	3.5 (2.1 to 5)	4.5 (2.5 to 6.7)	-16.5 (-39.9 to 30.2)	-19.2 (-46.5 to 33.7)	-14.9 (-47.3 to 46.4)
Kermanshah	Incidence	11.5 (8.1 to 14.6)	10.6 (6.9 to 14.1)	12.2 (7.5 to 16.5)	8.6 (6.3 to 10.5)	7.1 (5.2 to 8.8)	10.1 (6.2 to 13.2)	-24.8 (-45.4 to 9)	-33.1 (-55 to 6.3)	-16.6 (-45.9 to 28.5)
	Prevalence	43.2 (27.4 to 58.9)	45 (24.5 to 68.5)	41.1 (21.9 to 59.5)	33.3 (22.4 to 42.3)	29 (19.5 to 38.1)	37.6 (20 to 51.4)	-22.9 (-49.4 to 31.6)	-35.5 (-62.1 to 27.9)	-8.6 (-48.2 to 63.1)
	Deaths	8.5 (6.4 to 10.5)	7 (4.9 to 9.1)	9.8 (6.7 to 13)	6.2 (4.7 to 7.5)	4.8 (3.7 to 5.9)	7.6 (5.1 to 9.8)	-27.1 (-44.1 to 0.7)	-31.4 (-50.2 to -1.4)	-22.9 (-47.4 to 19)
	DALYs	335.3 (238.6 to 434.2)	303.9 (185.2 to 415)	361.2 (201.3 to 501.2)	218.7 (159.1 to 269.3)	173.8 (124.3 to 216.8)	262.8 (167.6 to 341.5)	-34.8 (-51.4 to -6.8)	-42.8 (-60.8 to -10.1)	-27.2 (-51.8 to 15.9)
	YLLs	330.1 (235.1 to 427.2)	298.9 (182.3 to 408.5)	355.7 (197.9 to 496)	214.4 (156.5 to 264.2)	170.3 (121.7 to 212.7)	257.9 (164 to 335.9)	-35 (-51.7 to -6.8)	-43 (-61.2 to -10.2)	-27.5 (-52 to 16)
	YLDs	5.3 (3.2 to 7.7)	5 (2.8 to 7.4)	5.5 (3.1 to 8.4)	4.2 (2.6 to 6.1)	3.6 (2.1 to 5)	4.9 (2.6 to 7.5)	-19.8 (-43.5 to 24.8)	-28.7 (-54.4 to 21.8)	-11.2 (-47.2 to 47.7)

Province	Measure	Age-standardized rate (per 100,000)						% Change (1990 to 2019)		
		1990			2019					
		Both	Female	Male	Both	Female	Male	Both	Female	Male
Khorasan-e-Razavi	Incidence	15 (9.4 to 20.4)	14.9 (7.9 to 21.2)	15.2 (6.8 to 21.9)	10.3 (6.6 to 12.9)	9.1 (5.7 to 11.4)	11.6 (6.4 to 15.5)	-31.3 (-50.8 to 2.5)	-39.4 (-60.3 to 3.1)	-23.3 (-50.6 to 23.6)
	Prevalence	60.4 (33.6 to 87.9)	67.8 (28.7 to 108.9)	53.2 (20.5 to 80.2)	40.8 (23.7 to 53.8)	37.9 (21.9 to 51)	43.9 (20.6 to 62)	-32.4 (-58.1 to 21.9)	-44.2 (-69.4 to 19.7)	-17.5 (-55.5 to 49.7)
	Deaths	10.6 (7.2 to 13.5)	9.2 (5.6 to 12)	12 (6.2 to 16.6)	7.4 (5 to 9)	6.1 (3.9 to 7.4)	8.8 (5.1 to 11.5)	-30.2 (-45.5 to -5.7)	-34.2 (-52.3 to -6.8)	-26.8 (-49 to 9.9)
	DALYs	440.4 (276.6 to 595.9)	422.4 (189.7 to 580.7)	456.8 (189.4 to 660.6)	254.3 (156.7 to 315.3)	217.1 (128.7 to 270.3)	291.6 (156.4 to 389.2)	-42.3 (-58.5 to -15.8)	-48.6 (-65.3 to -13.9)	-36.2 (-59.4 to 7.1)
	YLLs	433.4 (273.4 to 587.6)	415.3 (185.8 to 571.5)	449.9 (186.5 to 650.7)	249.2 (153.8 to 309.6)	212.6 (124.9 to 265.7)	286 (152.7 to 381.8)	-42.5 (-58.7 to -16.4)	-48.8 (-65.6 to -13.7)	-36.4 (-59.4 to 6.7)
	YLDs	7 (4 to 10.5)	7.1 (3.5 to 10.9)	6.9 (2.9 to 10.9)	5.1 (2.9 to 7.5)	4.6 (2.6 to 6.7)	5.6 (2.7 to 8.8)	-27 (-49.5 to 17.2)	-35.5 (-60.7 to 16.2)	-18.3 (-51.3 to 40)
Khuzestan	Incidence	8.7 (6.4 to 10.8)	7.9 (5.7 to 10.3)	9.5 (6.5 to 12.5)	7.9 (6.3 to 9.3)	6.8 (5.4 to 8.3)	9 (6.3 to 11.2)	-9.1 (-30 to 27.3)	-13.7 (-39.7 to 27.4)	-5.8 (-34 to 42.1)
	Prevalence	31.8 (21.8 to 41.5)	31.3 (20.4 to 44.7)	32.5 (19.7 to 44.9)	31.6 (23.7 to 38.9)	29.4 (21.9 to 38)	33.9 (20.6 to 44.4)	-0.6 (-28.6 to 60.5)	-5.9 (-40.3 to 56.9)	4.2 (-35.3 to 75.8)
	Deaths	6.7 (5.2 to 8)	5.6 (4.2 to 7.3)	7.8 (5.6 to 10.1)	5.5 (4.6 to 6.5)	4.4 (3.6 to 5.4)	6.7 (4.8 to 8.4)	-16.8 (-34.8 to 10.7)	-21.4 (-42.4 to 9.5)	-14.5 (-38.4 to 26.8)
	DALYs	246.3 (187 to 306.2)	220.7 (154.1 to 296.2)	271.3 (179.8 to 353.6)	191.7 (153.7 to 224.1)	160.1 (127.2 to 193.2)	223.3 (151.8 to 278.3)	-22.2 (-40.4 to 7.2)	-27.5 (-47.8 to 7.3)	-17.7 (-40.5 to 21.7)
	YLLs	242.3 (184.2 to 301.5)	217.1 (151 to 291.3)	266.9 (176.3 to 348.7)	187.7 (150.4 to 219.5)	156.6 (124.5 to 189.4)	218.8 (149.4 to 272.5)	-22.5 (-40.8 to 7)	-27.9 (-48.2 to 7.4)	-18 (-40.7 to 21.6)
	YLDs	4 (2.5 to 5.7)	3.7 (2.2 to 5.4)	4.4 (2.5 to 6.5)	4 (2.6 to 5.6)	3.5 (2.3 to 5)	4.4 (2.6 to 6.5)	-0.8 (-26.1 to 45.3)	-4.7 (-35.8 to 44.2)	1.6 (-33.6 to 61.1)

Province	Measure	Age-standardized rate (per 100,000)						% Change (1990 to 2019)		
		1990			2019					
		Both	Female	Male	Both	Female	Male	Both	Female	Male
Kohgiluyeh and Boyer-Ahmad	Incidence	13.1 (7.5 to 18.5)	12.6 (6.8 to 19.7)	13.6 (6.7 to 20)	10 (6.4 to 12.5)	8.5 (5.9 to 11)	11.2 (5.9 to 15.4)	-24 (-47.4 to 21.9)	-32.7 (-59.2 to 18.2)	-17.4 (-45.9 to 48.3)
	Prevalence	57.4 (26.3 to 90.4)	60.4 (25.2 to 108.3)	54.4 (22.5 to 87)	44.5 (27.3 to 57.9)	41 (27 to 55.9)	47.6 (23.4 to 67)	-22.5 (-53.1 to 60)	-32.1 (-64.7 to 57.9)	-12.5 (-49.3 to 84.4)
	Deaths	8.6 (5.4 to 11.4)	7.4 (4.6 to 9.7)	9.9 (5.5 to 14.4)	6.2 (4.1 to 7.8)	4.7 (3.4 to 5.9)	7.4 (4.1 to 10.1)	-28.7 (-46.4 to 2.6)	-36.8 (-55.3 to -4.2)	-25.1 (-49.5 to 17.4)
	DALYs	360.4 (210.7 to 502.7)	334.1 (177.3 to 481.6)	384.9 (187.2 to 574.6)	225.5 (144.1 to 283.8)	185.7 (121.7 to 233.8)	262.3 (140.1 to 359)	-37.4 (-55.1 to -3.7)	-44.4 (-62.9 to -5.9)	-31.9 (-54.3 to 14.1)
	YLLs	354.1 (207.2 to 492.8)	328 (174.1 to 472.6)	378.4 (183.8 to 567)	220.4 (141.3 to 277.6)	181.2 (117.9 to 228.8)	256.5 (137 to 351.2)	-37.8 (-55.4 to -4.5)	-44.8 (-63 to -6.1)	-32.2 (-54.6 to 13.2)
	YLDs	6.3 (3.1 to 10.2)	6.1 (2.9 to 10.4)	6.5 (2.9 to 11.1)	5.2 (3 to 7.8)	4.5 (2.7 to 6.6)	5.8 (2.8 to 9)	-17.8 (-45.4 to 44.5)	-26.1 (-57.8 to 41.9)	-11.5 (-43.9 to 67)
Kurdistan	Incidence	14.1 (9.1 to 20.3)	14.8 (8 to 23.4)	13.3 (7.3 to 19.4)	8.5 (5.7 to 10.6)	6.9 (4.3 to 8.6)	10.1 (6 to 13.3)	-39.9 (-60 to -4.5)	-53.6 (-72.5 to -11.9)	-24.4 (-52.5 to 24.6)
	Prevalence	62.5 (32 to 99.6)	74.5 (29.7 to 135.4)	50.5 (22.3 to 79.8)	33.5 (20.5 to 44.2)	28.6 (16.1 to 39.8)	38.4 (19.7 to 53.3)	-46.3 (-68.9 to 8.6)	-61.6 (-81 to -3.1)	-24 (-59.2 to 46.2)
	Deaths	9.2 (6.6 to 11.8)	8.1 (5.1 to 10.6)	10.1 (6.4 to 13.9)	6 (4.1 to 7.3)	4.6 (3.1 to 5.5)	7.4 (4.4 to 9.6)	-34.1 (-50.9 to -10.4)	-43 (-59.3 to -16.3)	-26.2 (-51.6 to 12.8)
	DALYs	390.4 (264.1 to 549.8)	393.7 (193.7 to 574.5)	383.2 (191.4 to 558.2)	208.2 (133 to 258.2)	159.8 (98.6 to 198.5)	255.6 (144.8 to 335.1)	-46.7 (-63.1 to -19.9)	-59.4 (-74.1 to -24.4)	-33.3 (-58.8 to 10.3)
	YLLs	383.7 (259.6 to 539.4)	386.6 (190.1 to 564.4)	377 (187.8 to 551.5)	204 (129.8 to 252.7)	156.3 (96.5 to 193.9)	250.7 (141.7 to 329.2)	-46.8 (-63.2 to -20.1)	-59.6 (-74.2 to -24.6)	-33.5 (-59 to 10.1)
	YLDs	6.7 (3.7 to 10.6)	7.1 (3.2 to 12.4)	6.2 (3.1 to 10)	4.2 (2.4 to 6.1)	3.5 (1.9 to 5.1)	4.9 (2.5 to 7.4)	-36.9 (-59.6 to 9)	-51.2 (-72.9 to -0.9)	-20.5 (-53.8 to 40)

Province	Measure	Age-standardized rate (per 100,000)						% Change (1990 to 2019)		
		1990			2019					
		Both	Female	Male	Both	Female	Male	Both	Female	Male
Lorestan	Incidence	9.3 (6.5 to 12.4)	8.6 (5.5 to 13)	9.9 (6.6 to 13.8)	7.4 (5.6 to 9)	5.4 (4 to 7)	9.4 (6.3 to 12)	-20.7 (-44.6 to 23.8)	-36.6 (-61.7 to 11.8)	-5.4 (-37 to 54.7)
	Prevalence	33.7 (20.1 to 51)	34.8 (17.7 to 61.4)	32.5 (18.7 to 48.9)	26.5 (19.1 to 34.2)	21 (13.9 to 28.6)	32.3 (19.2 to 44.4)	-21.4 (-52 to 52.7)	-39.7 (-70.3 to 40.6)	-0.5 (-42.9 to 92.3)
	Deaths	7.1 (5.3 to 8.8)	5.8 (4.2 to 7.6)	8.2 (5.6 to 10.8)	5.5 (4.3 to 6.6)	3.6 (2.6 to 4.5)	7.4 (5.1 to 9.4)	-22.7 (-41.5 to 11.8)	-38 (-58 to -7.4)	-10.1 (-37.7 to 43)
	DALYs	267 (192 to 361.4)	242.7 (149.9 to 351.3)	287.5 (181.1 to 404)	169.7 (127.1 to 208)	124.2 (84.1 to 160)	216.5 (142 to 275.3)	-36.4 (-55.3 to -3.2)	-48.8 (-68.4 to -12.2)	-24.7 (-50.5 to 21.3)
	YLLs	262.9 (189.7 to 354.4)	238.9 (148 to 345.4)	283.2 (177.6 to 398.1)	166.2 (124 to 203.8)	121.6 (82.5 to 157.5)	212.1 (139 to 269.7)	-36.8 (-55.5 to -3.7)	-49.1 (-68.6 to -12.7)	-25.1 (-50.6 to 20.6)
	YLDs	4.1 (2.4 to 6)	3.8 (2.1 to 6.1)	4.3 (2.4 to 6.6)	3.5 (2.3 to 4.9)	2.6 (1.5 to 3.8)	4.4 (2.6 to 6.3)	-15.2 (-42.7 to 46.2)	-32.5 (-62 to 31.8)	1.8 (-36.3 to 79.7)
Markazi	Incidence	13.4 (8.3 to 18.2)	12.4 (7 to 17.4)	14.4 (7.2 to 20.9)	9 (5.5 to 11.4)	7.5 (4.5 to 9.6)	10.6 (5.3 to 14.3)	-32.7 (-52.8 to 0.3)	-39.5 (-60.7 to 3.2)	-26.8 (-53.7 to 20.1)
	Prevalence	53 (28.9 to 76.7)	54.9 (25.7 to 85.8)	51.3 (22.3 to 77.8)	37 (20.2 to 49.1)	32.8 (18.1 to 45.1)	41.3 (18 to 57.8)	-30.3 (-54.7 to 21.8)	-40.3 (-66.1 to 20.5)	-19.5 (-54.5 to 48.3)
	Deaths	9.7 (6.3 to 12.7)	8 (4.9 to 10.4)	11.5 (6.3 to 16.1)	6.3 (4.1 to 7.8)	4.8 (3.1 to 5.9)	7.7 (4.1 to 10.4)	-35.4 (-50.8 to -11.1)	-39.6 (-56.8 to -11.8)	-32.4 (-55.1 to 6.4)
	DALYs	376.1 (240.6 to 505.1)	342 (180.1 to 465.8)	409.8 (196.4 to 584.5)	215.5 (134.3 to 272.2)	175.4 (102.3 to 222.9)	255.7 (132.8 to 347.6)	-42.7 (-58.7 to -18.5)	-48.7 (-65.1 to -15.4)	-37.6 (-60.3 to 2.8)
	YLLs	369.9 (235.9 to 496.9)	336.1 (176.4 to 457.7)	403.2 (193.2 to 577.3)	210.9 (131.6 to 266.2)	171.5 (100.5 to 218.8)	250.4 (130.2 to 339.5)	-43 (-58.8 to -18.8)	-49 (-65.2 to -15.6)	-37.9 (-60.6 to 2.6)
	YLDs	6.3 (3.4 to 9.4)	5.9 (3.1 to 9.2)	6.6 (3 to 10.6)	4.6 (2.4 to 6.9)	3.9 (2 to 5.7)	5.3 (2.3 to 8.3)	-27.4 (-50.5 to 15)	-35 (-59.9 to 14.6)	-20.6 (-52.8 to 33.7)

Province	Measure	Age-standardized rate (per 100,000)						% Change (1990 to 2019)		
		1990			2019					
		Both	Female	Male	Both	Female	Male	Both	Female	Male
Mazandaran	Incidence	10.6 (6.9 to 13.9)	10.4 (6 to 13.8)	10.9 (6.1 to 15.1)	9.7 (6.5 to 11.9)	8.7 (5.4 to 10.9)	10.8 (6 to 14.3)	-8.5 (-30.8 to 27.1)	-16.5 (-42.4 to 23.4)	-1.5 (-31.9 to 50.2)
	Prevalence	42.3 (24.6 to 58.6)	44.2 (22.1 to 63)	40.8 (19.2 to 61.5)	44.9 (27.7 to 56.6)	42.6 (24.6 to 55.9)	47.4 (22.9 to 66)	6.2 (-26.3 to 69)	-3.6 (-38.9 to 67.7)	16.3 (-25.2 to 105.4)
	Deaths	7.7 (5.3 to 10)	7 (4.5 to 9.3)	8.6 (5.2 to 12.1)	6 (4.3 to 7.4)	4.9 (3.3 to 6.1)	7.2 (4.4 to 9.3)	-22 (-39.2 to 7.5)	-29.8 (-48.4 to 1.3)	-16.6 (-41.1 to 23.2)
	DALYs	283.8 (184.6 to 366.9)	271.2 (150.5 to 370.8)	297.2 (162.9 to 418.1)	203.9 (139 to 250.4)	175.6 (108.1 to 220.8)	232.4 (135.5 to 304.9)	-28.1 (-44.9 to 1.9)	-35.2 (-53.4 to -1.8)	-21.8 (-44.3 to 20.7)
	YLLs	278.7 (180.3 to 360.5)	266.1 (146.9 to 364.1)	292.1 (160.2 to 412.4)	198.7 (135.4 to 244.3)	170.9 (103.8 to 214.5)	226.7 (133 to 297.6)	-28.7 (-45.4 to 1.4)	-35.8 (-53.9 to -2.2)	-22.4 (-45.1 to 19.5)
	YLDs	5.1 (2.8 to 7.7)	5 (2.5 to 7.6)	5.2 (2.5 to 8.4)	5.2 (3.1 to 7.4)	4.7 (2.5 to 6.8)	5.7 (2.7 to 8.7)	2.4 (-25.2 to 51.4)	-6.5 (-37.1 to 47.8)	10 (-27.4 to 80.9)
North Khorasan	Incidence	12.1 (7.9 to 17.6)	12.2 (7.3 to 19.5)	12 (7 to 17.2)	8.5 (6.6 to 9.9)	7.5 (5.8 to 9.1)	9.4 (6.5 to 11.6)	-30.1 (-53.3 to 10.2)	-38.6 (-64.2 to 3.9)	-21.4 (-47.9 to 33.2)
	Prevalence	50.5 (25.5 to 81.3)	56.7 (25.4 to 103.7)	44.4 (21.4 to 68.3)	34.5 (23.7 to 43)	32 (22.3 to 41.5)	37 (22.6 to 47.8)	-31.8 (-59 to 38.4)	-43.6 (-71.5 to 28.5)	-16.7 (-52 to 70.8)
	Deaths	8.5 (6.3 to 10.8)	7.6 (5.1 to 10.2)	9.3 (6.1 to 12.6)	5.9 (4.9 to 6.8)	4.9 (4 to 5.9)	6.8 (5 to 8.2)	-30.5 (-47.4 to -1.6)	-35.4 (-54.8 to -1.8)	-26.5 (-48.1 to 14.3)
	DALYs	347.7 (247.8 to 486.5)	340.9 (197.6 to 508.2)	352.6 (195.3 to 512)	208.6 (164.9 to 242.9)	181.4 (140 to 217.4)	235.7 (163.7 to 287.4)	-40 (-58.8 to -9.9)	-46.8 (-66 to -10.8)	-33.2 (-55.5 to 12.7)
	YLLs	341.9 (243.7 to 478.4)	335 (194.9 to 499)	347 (192 to 504.4)	204.3 (162.3 to 237.8)	177.6 (138.1 to 213.3)	231 (161 to 282.8)	-40.3 (-59 to -10.4)	-47 (-66.1 to -11.3)	-33.4 (-55.9 to 12.7)
	YLDs	5.7 (3.2 to 9)	5.9 (2.9 to 9.8)	5.6 (2.9 to 8.8)	4.3 (2.8 to 6)	3.8 (2.4 to 5.4)	4.7 (2.8 to 6.9)	-25.3 (-51.2 to 28.9)	-34.5 (-62.6 to 22.5)	-15.7 (-47.7 to 55.6)

Province	Measure	Age-standardized rate (per 100,000)						% Change (1990 to 2019)		
		1990			2019					
		Both	Female	Male	Both	Female	Male	Both	Female	Male
Qazvin	Incidence	10.4 (7.1 to 14.2)	10.4 (6.2 to 15.5)	10.5 (6.5 to 14.3)	8 (5.7 to 9.8)	6 (4 to 7.4)	10.2 (6.3 to 13.1)	-23.1 (-45.4 to 14.4)	-42.5 (-65.2 to -1.2)	-3.5 (-34.9 to 57.7)
	Prevalence	42 (24.3 to 63.9)	46.9 (22.2 to 82)	37.5 (18.6 to 55.7)	31.7 (20.5 to 40.7)	25.4 (15.2 to 33.8)	38.5 (21.2 to 51.7)	-24.4 (-53.1 to 39.5)	-45.8 (-72.2 to 20.9)	2.6 (-38.3 to 94.8)
	Deaths	7.4 (5.4 to 9.3)	6.5 (4.3 to 8.3)	8.4 (5.5 to 11.3)	5.7 (4.3 to 6.9)	3.8 (2.9 to 4.7)	7.8 (5.1 to 9.8)	-22.6 (-40.5 to 9.4)	-40.9 (-57.4 to -12.5)	-7.7 (-35.6 to 42.3)
	DALYs	286.6 (198.9 to 383.2)	277.3 (164.7 to 383.7)	295.8 (169 to 411.3)	179 (126.3 to 220.1)	133.4 (90.5 to 162.7)	226.2 (139.5 to 290.1)	-37.5 (-54.3 to -8.2)	-51.9 (-68.6 to -21.2)	-23.6 (-49.9 to 23.9)
	YLLs	281.7 (195.6 to 376.4)	272.4 (162.1 to 376.6)	290.9 (166.6 to 405)	174.9 (123.1 to 215.1)	130.3 (88.7 to 160.3)	221 (136.5 to 285.2)	-37.9 (-54.7 to -8.6)	-52.1 (-68.7 to -21.1)	-24 (-50.3 to 23.3)
	YLDs	4.9 (2.8 to 7.5)	4.9 (2.5 to 8)	4.9 (2.6 to 7.6)	4.1 (2.5 to 5.8)	3.1 (1.7 to 4.3)	5.1 (2.8 to 7.7)	-16.8 (-42.9 to 35.3)	-38.2 (-63.3 to 13.5)	5.1 (-32 to 83.8)
Qom	Incidence	13.5 (7.1 to 18.2)	13.5 (6.3 to 19.3)	13.5 (6.5 to 19.8)	9.6 (5.3 to 12.1)	8.1 (4.4 to 10.1)	11 (4.9 to 14.9)	-28.7 (-49.5 to 13.9)	-40.1 (-61.5 to 7.5)	-18.2 (-47 to 44.7)
	Prevalence	50.8 (23.7 to 76.4)	55.9 (21.9 to 91.2)	46 (19.6 to 71.2)	38.8 (18.5 to 51.6)	33.7 (16.4 to 45.1)	43.7 (16.3 to 60.6)	-23.5 (-50.3 to 40.7)	-39.7 (-66.2 to 29.5)	-5.1 (-45.6 to 90.5)
	Deaths	10.2 (5.7 to 13.6)	9.4 (4.7 to 12.4)	11 (5.5 to 16)	6.9 (4.1 to 8.6)	5.6 (3.4 to 7)	8 (3.8 to 10.8)	-32.9 (-48.8 to 1.9)	-40.9 (-57.7 to -1.9)	-27.2 (-51.1 to 20.2)
	DALYs	377.2 (197.4 to 512.2)	366.6 (161.7 to 507.1)	387.5 (173.5 to 578)	216.7 (120.1 to 272.3)	174.1 (94 to 218.7)	257.3 (114.2 to 347.2)	-42.6 (-58.4 to -6.7)	-52.5 (-68.2 to -7.6)	-33.6 (-57.1 to 25.5)
	YLLs	370.9 (193.9 to 503.6)	360.2 (158 to 500)	381.3 (170.3 to 569.3)	211.8 (117.5 to 266.5)	169.9 (92.2 to 213.1)	251.6 (111.8 to 339)	-42.9 (-58.8 to -7.2)	-52.8 (-68.3 to -7.5)	-34 (-57.4 to 24.6)
	YLDs	6.3 (2.9 to 9.7)	6.4 (2.8 to 9.8)	6.2 (2.8 to 10.3)	4.9 (2.4 to 7.3)	4.2 (1.9 to 6.1)	5.6 (2.2 to 8.8)	-21.2 (-44.7 to 33.8)	-34.6 (-60.3 to 25.7)	-8.5 (-44.5 to 68)

Province	Measure	Age-standardized rate (per 100,000)						% Change (1990 to 2019)		
		1990			2019					
		Both	Female	Male	Both	Female	Male	Both	Female	Male
Semnan	Incidence	10.9 (6.9 to 14.2)	9.3 (5.6 to 12.5)	12.6 (6.9 to 17.8)	8.8 (5.9 to 10.9)	6.4 (4.5 to 8.1)	11.3 (6.5 to 14.5)	-19.2 (-40.5 to 20.8)	-30.9 (-52.2 to 8.9)	-10.9 (-41 to 39.8)
	Prevalence	44 (23.2 to 62.3)	40.5 (20.8 to 61.8)	47.9 (22.4 to 71.5)	38.5 (23.5 to 49.7)	29.9 (18.3 to 40.3)	47.4 (24.3 to 63.9)	-12.6 (-41.2 to 53.2)	-26.2 (-55.9 to 41.5)	-1.1 (-42.5 to 77)
	Deaths	8 (5.5 to 10.3)	6.3 (4.1 to 8.2)	10 (5.9 to 13.7)	5.8 (4.2 to 7.1)	3.9 (2.9 to 4.7)	8 (4.8 to 10.2)	-26.7 (-44.2 to 4.9)	-38.4 (-55.3 to -11.7)	-19.6 (-43.8 to 28.5)
	DALYs	293.2 (191.5 to 388.5)	249.1 (152.1 to 323.9)	338.6 (177.3 to 484.5)	182.6 (124.1 to 225.9)	133.8 (90.2 to 163.6)	232.7 (132.9 to 303.8)	-37.7 (-54.1 to -9.8)	-46.3 (-61.5 to -17.4)	-31.3 (-53.4 to 14.4)
	YLLs	287.9 (188.3 to 381.5)	244.6 (148.8 to 318.9)	332.5 (173.7 to 476.7)	178 (121.4 to 221.4)	130.4 (88.3 to 158.7)	226.7 (129.5 to 296.5)	-38.2 (-54.4 to -10.5)	-46.7 (-62 to -18)	-31.8 (-53.9 to 13.9)
	YLDs	5.3 (2.8 to 7.8)	4.5 (2.4 to 6.8)	6.1 (2.9 to 9.6)	4.7 (2.6 to 6.8)	3.4 (2 to 5)	6 (2.9 to 9.1)	-11.3 (-36.5 to 40.6)	-24 (-49.2 to 30.1)	-2.3 (-38.6 to 67)
Sistan and Baluchistan	Incidence	7.6 (3.9 to 10.7)	6.7 (3.5 to 9.9)	8.3 (4.3 to 12.5)	5.7 (4.7 to 7.8)	4.7 (3.7 to 7)	6.7 (5.2 to 9.5)	-24.1 (-47.4 to 43)	-29.9 (-55.5 to 31.9)	-18.5 (-45.6 to 57.1)
	Prevalence	26.5 (11.7 to 41)	27 (11.2 to 46.7)	25.6 (12.3 to 41.9)	18.2 (14.7 to 24.4)	16.4 (12.2 to 25.6)	19.9 (15.2 to 26.9)	-31.4 (-56.8 to 50.8)	-39.1 (-66.7 to 47.7)	-22.6 (-54 to 65.5)
	Deaths	5.8 (3.4 to 8.1)	4.6 (2.8 to 7)	6.9 (3.8 to 10.5)	4.6 (3.7 to 6.2)	3.5 (2.7 to 5.5)	5.6 (4.2 to 8.2)	-21.7 (-41.7 to 30.5)	-25.2 (-48.5 to 28.3)	-18.3 (-44.5 to 43.7)
	DALYs	234.6 (109.2 to 325.3)	202.1 (87.4 to 290.2)	262.4 (120.8 to 390.1)	167.4 (139.8 to 214.8)	135.6 (109.2 to 193)	198.5 (157.2 to 269.1)	-28.7 (-50.9 to 48.1)	-32.9 (-56.6 to 56.1)	-24.4 (-51.2 to 64.9)
	YLLs	231.3 (107.4 to 321.3)	199.1 (85.9 to 286.6)	258.8 (119.1 to 384.7)	164.9 (137.4 to 212.3)	133.5 (107.1 to 189.4)	195.6 (154.7 to 264.8)	-28.7 (-51 to 48.2)	-33 (-56.8 to 56.4)	-24.4 (-51.5 to 65.5)
	YLDs	3.3 (1.5 to 5.1)	3 (1.3 to 4.9)	3.5 (1.6 to 5.8)	2.5 (1.7 to 3.6)	2.1 (1.4 to 3.4)	2.8 (1.8 to 4.3)	-24.6 (-49 to 42.6)	-29.4 (-56.7 to 44.5)	-19.7 (-49 to 55.7)

Province	Measure	Age-standardized rate (per 100,000)						% Change (1990 to 2019)		
		1990			2019					
		Both	Female	Male	Both	Female	Male	Both	Female	Male
South Khorasan	Incidence	13 (7.7 to 18.8)	11.9 (6.2 to 18.7)	14 (7.2 to 21)	8.4 (5.5 to 10.3)	6.7 (4.5 to 8.4)	10.2 (5.4 to 13.3)	-35.3 (-55.9 to 2.1)	-43.8 (-66.3 to -4.1)	-27 (-54.9 to 27.2)
	Prevalence	54.4 (24.4 to 87.6)	53.5 (20.3 to 96.9)	55.1 (22.7 to 90.4)	32.2 (18.7 to 41.8)	26 (15.6 to 35.1)	38.5 (18.1 to 52.5)	-40.8 (-64.3 to 21.8)	-51.4 (-75.6 to 16.3)	-30 (-63.8 to 45.7)
	Deaths	9 (6 to 12.1)	7.5 (4.5 to 10)	10.4 (6.1 to 15.2)	6.1 (4.3 to 7.3)	4.6 (3 to 5.5)	7.7 (4.5 to 9.9)	-32.1 (-49.3 to -6.4)	-38.9 (-57.2 to -9.5)	-25.7 (-49.2 to 15)
	DALYs	365 (229.5 to 528.5)	329.6 (169.7 to 475.3)	397.6 (190.1 to 626.3)	205.2 (134.8 to 249.6)	164.4 (100.7 to 200)	246.7 (137.2 to 314.5)	-43.8 (-61.2 to -17.6)	-50.1 (-67.2 to -17.4)	-37.9 (-60.5 to 6.1)
	YLLs	359 (225 to 520)	324.2 (166.9 to 467.6)	391 (186.4 to 617.4)	201.2 (132.4 to 245)	161.3 (98.8 to 196.6)	241.8 (134.4 to 309.5)	-44 (-61.4 to -17.8)	-50.2 (-67.2 to -18)	-38.2 (-60.9 to 6.5)
	YLDs	6 (2.9 to 9.8)	5.4 (2.5 to 9.4)	6.6 (3 to 11)	4 (2.2 to 5.8)	3.1 (1.8 to 4.5)	4.9 (2.3 to 7.4)	-33.7 (-56.8 to 15.6)	-43 (-67.6 to 8.1)	-25 (-56.1 to 36.7)
Tehran	Incidence	10.9 (5.5 to 16.2)	11.6 (6.1 to 17.8)	10.4 (4.3 to 15.9)	8.1 (4.5 to 9.9)	7.5 (4.3 to 9.4)	8.7 (3.3 to 11.5)	-26 (-48.7 to 7.3)	-35.7 (-60.2 to 4.4)	-16.1 (-45.2 to 26.2)
	Prevalence	49.9 (20.4 to 82.7)	57.3 (23.6 to 103.3)	43 (16.1 to 72.4)	37.5 (18.1 to 47.9)	35.9 (17.6 to 47.7)	39 (12.5 to 55.1)	-24.8 (-52.9 to 29.6)	-37.3 (-67.1 to 30.2)	-9.2 (-46.3 to 54.5)
	Deaths	7.2 (4.1 to 9.6)	6.9 (4.1 to 9.5)	7.5 (3.6 to 10.9)	5 (2.9 to 6.1)	4.4 (2.7 to 5.4)	5.6 (2.4 to 7.3)	-30.6 (-47.5 to -7.4)	-36.5 (-55.3 to -11.5)	-25.9 (-48.7 to 9.2)
	DALYs	278.2 (148.7 to 396.7)	273.3 (150.3 to 399.2)	283.6 (126 to 427.8)	174.7 (93.5 to 215.4)	151 (87 to 183.1)	197.7 (77.1 to 259.9)	-37.2 (-55.1 to -11.8)	-44.7 (-63.4 to -16.2)	-30.3 (-53.4 to 8.2)
	YLLs	272.7 (146.3 to 387.9)	267.4 (148 to 389.7)	278.5 (124.2 to 420.4)	170.3 (91.7 to 210.1)	147 (85.2 to 178)	193 (74.9 to 254.1)	-37.5 (-55.5 to -12.3)	-45 (-63.6 to -16.5)	-30.7 (-53.7 to 8.7)
	YLDs	5.5 (2.5 to 8.9)	6 (2.6 to 9.9)	5.2 (1.9 to 9)	4.4 (2.1 to 6.3)	4.1 (1.9 to 6)	4.6 (1.6 to 7.2)	-21.2 (-46.2 to 18.8)	-31.6 (-59.1 to 23.1)	-10.2 (-43.5 to 44.9)

Province	Measure	Age-standardized rate (per 100,000)						% Change (1990 to 2019)		
		1990			2019					
		Both	Female	Male	Both	Female	Male	Both	Female	Male
West Azarbayejan	Incidence	12.4 (8.7 to 16.2)	12.1 (7.8 to 16.2)	12.7 (7.3 to 17.5)	9.3 (6.4 to 11.3)	8.1 (5.3 to 10.1)	10.6 (6.3 to 13.6)	-25 (-44.2 to 6.8)	-33.2 (-54.3 to 4.1)	-16.4 (-43.4 to 26.8)
	Prevalence	47.8 (30.3 to 66.7)	52.3 (27.9 to 79.3)	43.3 (20.9 to 64.3)	36 (22.3 to 46.4)	33.4 (20.7 to 45.2)	38.9 (19.9 to 52.1)	-24.6 (-51 to 28.5)	-36.1 (-63.4 to 23)	-10.1 (-47.9 to 59)
	Deaths	9.1 (6.7 to 11.2)	7.8 (5.7 to 10)	10.3 (6.3 to 13.6)	6.9 (5 to 8.2)	5.5 (4 to 6.6)	8.3 (5.4 to 10.7)	-24.3 (-40 to -0.9)	-29.9 (-47.9 to -1.8)	-19.1 (-40.8 to 16.3)
	DALYs	361 (254.2 to 476.4)	346.3 (210.3 to 469.1)	374 (198.7 to 519.9)	226.2 (154.6 to 273.3)	195.5 (127.5 to 237.9)	258.2 (157.5 to 329.3)	-37.4 (-53.5 to -14)	-43.5 (-61.2 to -11)	-31 (-51.8 to 7)
	YLLs	355.3 (249.4 to 468.1)	340.6 (207.3 to 460.3)	368.2 (195.4 to 511.9)	221.5 (151.8 to 268.2)	191.4 (124.9 to 233.6)	253 (155.4 to 324.4)	-37.7 (-53.8 to -14.2)	-43.8 (-61.3 to -11.2)	-31.3 (-52.2 to 6.8)
	YLDs	5.7 (3.5 to 8.3)	5.6 (3.2 to 8.6)	5.8 (3 to 8.9)	4.6 (2.7 to 6.4)	4.1 (2.4 to 5.8)	5.2 (2.8 to 7.7)	-19.1 (-42.1 to 22.5)	-27.9 (-53.9 to 20.9)	-9.9 (-42.9 to 44.3)
Yazd	Incidence	11.9 (7 to 15.5)	12.3 (6 to 16.3)	11.9 (6.6 to 16.5)	10.9 (6.6 to 13.6)	10.2 (5.4 to 13.4)	11.7 (6.4 to 16)	-8.4 (-30.2 to 35.2)	-16.4 (-41.4 to 32)	-1.6 (-34.3 to 59.8)
	Prevalence	45.9 (24.4 to 63.1)	50.5 (21.4 to 73.2)	42 (21 to 60.3)	49.2 (26.4 to 64.4)	49.1 (24.5 to 68.5)	49.5 (24.4 to 70.9)	7.4 (-23.5 to 79.6)	-2.8 (-39.4 to 80.5)	17.9 (-26.5 to 108.9)
	Deaths	9 (5.7 to 11.7)	8.6 (4.6 to 11.8)	9.7 (5.6 to 13.4)	7 (4.5 to 8.8)	6 (3.4 to 7.6)	8.2 (4.7 to 11)	-22.3 (-39.5 to 7.7)	-30.6 (-50.6 to 1.5)	-16.3 (-41.6 to 29.3)
	DALYs	324 (195.2 to 417.8)	331 (158.6 to 443.4)	321.1 (173.2 to 452.3)	228.6 (137.7 to 286.2)	208.8 (108.6 to 263)	248.4 (136.6 to 336.9)	-29.4 (-46.2 to 0.3)	-36.9 (-54 to -3)	-22.6 (-47.5 to 22.5)
	YLLs	318.4 (192.1 to 411.3)	325.2 (155.8 to 435.7)	315.5 (169.9 to 445.7)	222.8 (133.9 to 278.8)	203.2 (106.3 to 257.4)	242.2 (132.9 to 329.1)	-30 (-46.9 to -0.7)	-37.5 (-54.5 to -3.4)	-23.2 (-48.1 to 21.8)
	YLDs	5.6 (2.9 to 8.3)	5.8 (2.6 to 8.6)	5.5 (2.8 to 8.6)	5.8 (3.1 to 8.5)	5.5 (2.6 to 8.2)	6.2 (2.9 to 9.8)	4.1 (-22.7 to 64.1)	-4.7 (-36 to 56.8)	11.3 (-30 to 90.4)

Province	Measure	Age-standardized rate (per 100,000)						% Change (1990 to 2019)		
		1990			2019					
		Both	Female	Male	Both	Female	Male	Both	Female	Male
Zanjan	Incidence	10.7 (7.4 to 14)	11.2 (6.5 to 15.9)	10.3 (6.2 to 14.1)	7.1 (5.2 to 8.7)	5.6 (3.6 to 7)	8.8 (5.5 to 11)	-33.2 (-51.9 to 1)	-50 (-67.5 to -11.2)	-14.6 (-43.1 to 41.9)
	Prevalence	45.2 (26.3 to 66.3)	53 (23.8 to 87.4)	37.8 (18.2 to 57.4)	29 (18.7 to 37.5)	24.3 (13.5 to 32.8)	34 (18.6 to 45.3)	-36 (-59.5 to 21.1)	-54.2 (-75.2 to 5.7)	-10 (-49.3 to 79.9)
	Deaths	7.2 (5.4 to 8.8)	6.6 (4.4 to 8.5)	8 (5.4 to 10.4)	5.1 (3.9 to 6)	3.6 (2.6 to 4.4)	6.7 (4.4 to 8.3)	-29.7 (-44.9 to -5.2)	-45.3 (-58.4 to -23.7)	-16.3 (-40.9 to 26)
	DALYs	294.9 (207.5 to 383)	299.2 (165.1 to 410.5)	290.8 (162.1 to 395.7)	158.9 (113.4 to 193.1)	123.9 (78.2 to 153.5)	195.7 (122.4 to 246.5)	-46.1 (-60.6 to -21.9)	-58.6 (-71.2 to -28.1)	-32.7 (-55.3 to 12.7)
	YLLs	289.8 (203.2 to 377.4)	293.8 (162.7 to 403.2)	286 (159.4 to 390.1)	155.3 (111.3 to 188.9)	121 (76.6 to 150.2)	191.2 (119.8 to 240.3)	-46.4 (-60.8 to -22.6)	-58.8 (-71.3 to -28.5)	-33.1 (-55.7 to 11.4)
	YLDs	5.1 (2.9 to 7.5)	5.4 (2.7 to 8.6)	4.8 (2.4 to 7.4)	3.7 (2.2 to 5.3)	2.9 (1.6 to 4.2)	4.5 (2.5 to 6.6)	-27.5 (-49.7 to 18.7)	-46.2 (-67.4 to 1.3)	-6.1 (-42.1 to 64.4)

Data in parentheses are 95% Uncertainty Intervals (95% UIs); DALYs= Disability-Adjusted Life Years; YLLs= Years of Life Lost; YLDs= Years Lived with Disability
