# Supplementary material for: National and subnational burden of leukemia and its risk factors, 1990–2019: Results from the Global Burden of Disease study 2019
Source: PLoS One. 2023 Nov 30;18(11):e0287917. doi: 10.1371/journal.pone.0287917 (PMC10688854; doi:10.1371/journal.pone.0287917)
Supplement: S2 Table — (PDF) [file pone.0287917.s002.pdf]

Supplementary Table 2. Decomposition analysis of incident cases of leukemia by sex at national and provincial levels

Location		Sex	New cases		Expected new cases in 2019		% 1990 - 2019 new cases change cause			% 1990 - 2019 new cases overall change
			1990	2019	Population growth	Population growth + Aging	Population growth	Age structure change	Incidence rate change	
**Iran (Islamic Republic of)**		Both	6,092	6,767	8,772	8,798	44%	0.4%	-33.3%	11.1%
		Female	3,197	2,923	4,634	4,286	44.9%	-10.9%	-42.6%	-8.6%
		Male	2,895	3,844	4,142	4,503	43.1%	12.5%	-22.8%	32.8%
**Subnational**	Alborz	Both	112	196	218	228	95.4%	8.7%	-28.8%	75.4%
		Female	57	81	113	113	99%	-0.1%	-56.1%	42.8%
		Male	55	115	106	116	92%	18.2%	-1.2%	109%
	Ardebil	Both	182	111	201	175	10.9%	-14.7%	-35.1%	-38.9%
		Female	100	44	111	89	10.9%	-21.9%	-44.7%	-55.6%
		Male	82	67	91	85	10.8%	-7.4%	-21.9%	-18.4%
	Bushehr	Both	73	100	125	126	71.4%	2.1%	-36.9%	36.7%
		Female	42	47	70	67	64.7%	-6.1%	-47.4%	11.2%
		Male	30	52	54	59	78%	15%	-20.7%	72.3%
	Chahar Mahaal and Bakhtiari	Both	72	80	97	98	35.3%	1.2%	-25.6%	10.9%
		Female	36	32	48	45	36%	-10.6%	-34.9%	-9.5%
		Male	36	48	49	53	34.8%	11.1%	-15%	30.8%
	East Azarbayejan	Both	473	382	559	536	18.2%	-4.8%	-32.7%	-19.3%
		Female	259	167	307	271	18.1%	-13.7%	-40.2%	-35.8%
		Male	214	215	253	263	18.2%	5%	-22.5%	0.7%
	Fars	Both	378	511	513	555	35.9%	11.1%	-11.6%	35.4%
		Female	180	209	245	243	36.5%	-1.2%	-18.9%	16.4%
		Male	198	302	268	311	35.4%	22%	-4.7%	52.7%
	Gilan	Both	207	265	231	282	11.6%	24.2%	-8.2%	27.7%
		Female	107	119	120	133	11.7%	12.1%	-12.9%	10.9%
		Male	100	146	112	150	11.4%	38.6%	-4.5%	45.5%
	Golestan	Both	124	133	178	178	43.9%	-0.1%	-36.3%	7.5%
		Female	67	58	97	90	44.3%	-9.7%	-47.6%	-12.9%
		Male	57	75	81	87	43.5%	10.3%	-22.3%	31.6%
	Hamadan	Both	226	171	234	226	3.4%	-3.3%	-24.5%	-24.4%
		Female	122	73	128	113	5.1%	-12.6%	-32.6%	-40.1%
		Male	104	98	106	112	1.8%	6.4%	-14.2%	-5.9%
	Hormozgan	Both	74	101	153	144	105%	-11.6%	-58.2%	35.2%
		Female	38	38	78	69	105.1%	-23%	-80.7%	1.4%
		Male	36	62	75	74	104.9%	-1.2%	-33.1%	70.5%

Location	Sex	New cases 1990	New cases 2019	Expected new cases in 2019: Population growth	Expected new cases in 2019: Population growth + Aging	% 1990 - 2019 new cases change cause: Population growth	% 1990 - 2019 new cases change cause: Age structure change	% 1990 - 2019 new cases change cause: Incidence rate change	% 1990 - 2019 new cases overall change
Ilam	Both	43	44	57	53	32%	-9.9%	-21.3%	0.8%
	Female	19	15	25	21	33.7%	-22.7%	-30.1%	-19.1%
	Male	24	28	32	31	30.4%	-3.1%	-10.8%	16.5%
Isfahan	Both	353	506	484	528	37.1%	12.5%	-6.2%	43.4%
	Female	176	219	246	244	39.7%	-1.5%	-14.2%	24%
	Male	177	287	238	286	34.6%	27.5%	0.6%	62.7%
Kerman	Both	165	232	294	305	78.3%	6.5%	-44.2%	40.6%
	Female	80	100	141	137	76%	-4.9%	-45.5%	25.5%
	Male	85	132	153	168	80.5%	17.2%	-42.8%	54.9%
Kermanshah	Both	170	163	198	212	16.3%	8.2%	-28.6%	-4.1%
	Female	85	67	100	96	18.7%	-4.7%	-34.3%	-20.3%
	Male	86	96	98	113	14.1%	18.2%	-20.4%	11.9%
Khorasan-e-Razavi	Both	704	616	996	924	41.4%	-10.1%	-43.8%	-12.6%
	Female	382	274	541	465	41.7%	-20%	-50%	-28.3%
	Male	322	342	454	456	41.1%	0.4%	-35.3%	6.1%
Khuzestan	Both	229	329	349	371	52.2%	9.7%	-18.3%	43.6%
	Female	111	144	170	172	53.2%	1.8%	-25.3%	29.7%
	Male	118	185	179	199	51.3%	17.1%	-11.8%	56.6%
Kohgiluyeh and Boyer-Ahmad	Both	65	63	99	85	51.9%	-21.7%	-34.6%	-4.4%
	Female	35	27	52	41	51.9%	-32.6%	-42.2%	-23%
	Male	31	36	47	44	52%	-8.9%	-26.6%	16.5%
Kurdistan	Both	179	129	240	210	33.8%	-16.4%	-45.2%	-27.8%
	Female	104	52	139	109	34.2%	-29.3%	-54.2%	-49.3%
	Male	76	77	101	100	33.5%	-0.8%	-31.1%	1.6%
Lorestan	Both	125	119	143	150	14.3%	5.2%	-25.1%	-5.5%
	Female	63	45	73	69	15.5%	-6.1%	-38.1%	-28.6%
	Male	62	73	70	79	13.2%	14.2%	-9.4%	18%
Markazi	Both	144	131	171	185	19.2%	9.6%	-37.5%	-8.7%
	Female	71	54	84	82	18.5%	-2.6%	-40.1%	-24.2%
	Male	73	77	87	102	19.9%	21.2%	-34.6%	6.5%
Mazandaran	Both	231	340	304	354	31.5%	21.9%	-6%	47.4%
	Female	119	153	156	173	31.3%	14.2%	-16.4%	29%
	Male	112	187	148	182	31.6%	30.8%	4.3%	66.8%

Location		Sex	New cases		Expected new cases in 2019		% 1990 - 2019 new cases change cause			% 1990 - 2019 new cases overall change
			1990	2019	Population growth	Population growth + Aging	Population growth	Age structure change	Incidence rate change	
	North Khorasan	Both	75	66	103	97	37.5%	-7.5%	-41.3%	-11.3%
		Female	41	29	57	49	37.6%	-18.5%	-47.8%	-28.6%
		Male	33	37	46	48	37.3%	4.8%	-32.1%	10%
	Qazvin	Both	87	96	122	122	39.9%	-0.6%	-29.8%	9.5%
		Female	47	37	66	60	40.4%	-12.1%	-50%	-21.7%
		Male	40	59	56	61	39.3%	12%	-5.8%	45.5%
	Qom	Both	83	111	153	159	84.1%	7.7%	-57.9%	33.9%
		Female	44	45	82	78	85.8%	-8%	-75.2%	2.6%
		Male	39	66	71	81	82.4%	25.8%	-39%	69.2%
	Semnan	Both	45	61	70	74	54.9%	7.8%	-28.3%	34.4%
		Female	20	23	32	31	57.1%	-1.2%	-42.3%	13.5%
		Male	25	38	39	43	52.9%	16.2%	-18.2%	50.9%
	Sistan and Baluchistan	Both	104	129	210	196	101.9%	-13.2%	-64.2%	24.5%
		Female	51	55	103	94	102%	-17.3%	-76.3%	8.5%
		Male	53	74	107	101	101.9%	-11.5%	-50.4%	39.9%
	South Khorasan	Both	90	65	112	102	24.2%	-11.4%	-41.2%	-28.4%
		Female	44	26	54	47	24.1%	-16.5%	-47.9%	-40.3%
		Male	47	39	58	54	24.4%	-7.9%	-33.6%	-17.2%
	Tehran	Both	851	1,059	1,395	1,388	63.8%	-0.7%	-38.7%	24.4%
		Female	467	483	782	718	67.3%	-13.6%	-50.4%	3.3%
		Male	384	576	617	675	60.5%	15.1%	-25.7%	49.9%
	West Azarbayejan	Both	266	276	391	381	46.6%	-3.7%	-39.5%	3.5%
		Female	142	123	209	189	46.8%	-13.8%	-46.5%	-13.6%
		Male	124	153	182	189	46.5%	5.8%	-29.4%	23%
	Yazd	Both	69	113	117	124	69.2%	10.9%	-16.5%	63.6%
		Female	37	53	63	64	70.3%	0.4%	-28.6%	42%
		Male	32	60	53	62	68.1%	25.7%	-5%	88.8%
	Zanjan	Both	91	72	110	105	21.6%	-5.9%	-36.6%	-21%
		Female	51	29	62	54	22.1%	-16.5%	-49.5%	-43.9%
		Male	39	43	48	51	21%	7.2%	-19.4%	8.8%
