# Supplementary material for: National and subnational burden of leukemia and its risk factors, 1990–2019: Results from the Global Burden of Disease study 2019
Source: PLoS One. 2023 Nov 30;18(11):e0287917. doi: 10.1371/journal.pone.0287917 (PMC10688854; doi:10.1371/journal.pone.0287917)
Supplement: Attachment — Submitted filename: 1.docx [file pone.0287917.s003.docx]

1-The trend analysis is a better topic for this investigation. The data that support the conclusions were not included in the manuscript.

2-The statistical models and methods that were implemented to analyze data are not appropriately selected. The multilevel analysis is a better model to analyze such data. It was not mentioned that the Bayesian and spatial models how were implemented, what were priors and posterior functions, how neighborhood structure was selected? How data were generated in the Bayesian method, … ?

3-There was not attached data to the manuscript.

4- The manuscript was presented in an intelligible and technical fashion, however, there is some wrong written phrases ant terms in Standard English, such as; it is showed; in the line 88 in the Introduction section.

5-The tables of the results are very large.

6-The results of the proposed statistical analysis and methods were not presented in tables.
