# Supplementary material for: National and subnational burden of leukemia and its risk factors, 1990–2019: Results from the Global Burden of Disease study 2019
Source: PLoS One. 2023 Nov 30;18(11):e0287917. doi: 10.1371/journal.pone.0287917 (PMC10688854; doi:10.1371/journal.pone.0287917)
Supplement: Attachment — Submitted filename: Response to editor.docx [file pone.0287917.s004.docx]

Response to editor

Dear editor

I hope this letter finds you well. We are grateful about your comments on our manuscript and appreciate your consideration on our manuscript:

1. We ensured that your manuscript meets PLOS ONE's style requirements.
2. The dataset used during the current study is available on the GBD Compare Tool website (https://vizhub.healthdata.org/gbd-compare/).
3. All figures were illustrated using R statistical packages v4.1.0 (http://www.r-project.org/). The map images in figure 1 were created using Open Street Map’s data (without the use of an Open Street Map tile), and the Open Street Map’s shapefile was used in R statistical software to prepare these figures. To respond to your valuable inquiry, we revised the figure caption in the submitted files as “Contains information from OpenStreetMap and OpenStreetMap Foundation, which is made available under the Open Database License”.
4. All the reviewers’ comments were appreciable and helped us to improve the manuscript. We tried our best to brought their thoughtful comments to the manuscript.

King Regards
