# Supplementary material for: National and subnational burden of leukemia and its risk factors, 1990–2019: Results from the Global Burden of Disease study 2019
Source: PLoS One. 2023 Nov 30;18(11):e0287917. doi: 10.1371/journal.pone.0287917 (PMC10688854; doi:10.1371/journal.pone.0287917)
Supplement: Attachment — Submitted filename: Response to editor.docx [file pone.0287917.s005.docx]

Dear reviewers,

We are grateful about your kind comments. And we did our best to make our article better based on your comments. Below you could find our answers:

*Reviewer #1: Most of the initial comments have not been addressed.
It is not yet clear on what is known about the burden of leukemia in Iran and why it was important to have their study conducted.*

In the previous revision we checked all the initial comments and in our manuscript with tracked changes you could find them all.

As we described in the manuscript, the leukemia burden in Iran was not assessed before and there were some disparities in different cities and provinces that should be consider to be changed. So firstly, it was necessary to address the burden of this disease in our country then with our applied statistics we will need to take action to overcome these disparities and also try to reduce the costs of treatments. *Data abstraction process is still not very clear. A paragraph in the method section talked about the use of systematic reviews but, It is not further shown how authors based their results/discussion on this.*

We used those systematic reviews to address the most common risk factors of leukemia in our country. And also we used published systematic reviews and meta-analyzed these relative risks to estimate relative risk as a function of exposure for each risk-outcome pair.  *The first paragraph of the strength and limitation section appears more of recommendation.*

Thanks for your precise consideration. There was a small mistake which we corrected and improved it.

*Still the conclusion is not purely in line with the results.*

Thanks we tried to improve it based on our results.

*Reviewer #2: The comments to the authors raised in the previous round of review were not addressed and sent to me.*

In the previous revision we checked all the initial comments and in our manuscript with tracked changes you could find them all.

*However, there are some grammatical and editing errors in the text, which the authors should correct, as follows:*

*1- Page 7, line 237, the phrase our stud must be the study or this study*

*2- Page 7, line 242, the phrase our results must be the results*

*3- The page of line 259 of the phrase we observed a significant decrease must be a significant decrease was observed.*

Thanks for addressing these errors, we corrected them all.

King regards.
